# Corn yield components can be stabilized via tillering in sub-optimal plant densities

**DOI:** 10.3389/fpls.2022.1047268

**Published:** 2023-01-06

**Authors:** Rachel L. Veenstra, Carlos D. Messina, Dan Berning, Lucas A. Haag, Paul Carter, Trevor J. Hefley, P. V. Vara Prasad, Ignacio A. Ciampitti

**Affiliations:** ^1^ Department of Agronomy, Kansas State University, Manhattan, KS, United States; ^2^ Horticultural Sciences Department, University of Florida, Gainesville, FL, United States; ^3^ Corteva Agriscience Agronomy Sciences, Johnston, IA, United States; ^4^ Northwest Research-Extension Center, Kansas State University, Colby, KS, United States; ^5^ Formerly Corteva Agriscience, Independent Agronomist, Clive, IA, United States; ^6^ Department of Statistics, Kansas State University, Manhattan, KS, United States

**Keywords:** crop plasticity, yield components, tillering, plant density, corn (maize)

## Abstract

**Introduction:**

Crop plasticity is fundamental to sustainability discussions in production agriculture. Modern corn (*Zea mays* L.) genetics can compensate yield determinants to a small degree, but plasticity mechanisms have been masked by breeder selection and plant density management preferences. While tillers are a well-known source of plasticity in cereal crops, the functional trade-offs of tiller expression to the hierarchical yield formation process in corn are unknown. This investigation aimed to further dissect the consequences of tiller expression on corn yield component determination and plasticity in a range of environments from two plant fraction perspectives – i) main stalks only, considering potential functional trade-offs due to tiller expression; and ii) comprehensive (main stalk plus tillers).

**Methods:**

This multi-seasonal study considered a dataset of 17 site-years across Kansas, United States. Replicated field trials evaluated tiller presence (removed or intact) in two hybrids (P0657AM and P0805AM) at three target plant densities (25000, 42000, and 60000 plants ha^-1^). Record of ears and kernels per unit area and kernel weight were collected separately for both main stalks and tillers in each plot.

**Results:**

Indicated tiller contributions impacted the plasticity of yield components in evaluated genotypes. Ear number and kernel number per area were less dependent on plant density, but kernel number remained key to yield stability. Although ear number was less related to yield stability, ear source and type were significant yield predictors, with tiller axillary ears as stronger contributors than main stalk secondary ears in high-yielding environments.

**Discussions:**

Certainly, managing for the most main stalk primary ears possible – that is, optimizing the plant density (which consequently reduces tiller expression), is desirable to maximize yields. However, the demonstrated escape from the deterministic hierarchy of corn yield formation may offer avenues to reduce corn management dependence on a seasonally variable optimum plant density, which cannot be remediated mid-season.

## 1 Introduction

Corn (*Zea mays* L.) is of significant global socioeconomic importance, experiencing recent production expansion into more restrictive environments with less opportunity for intensified farming systems (Lark et al., 2020). In these regions of reduced yield potential, such as the Central High Plains of the United States (US), effective resource use is a key factor considered by farmers as they adapt to variable climatic conditions (Lobell et al., 2011). While farming systems can be acclimated to environmental conditions through management practices, such strategies often must be implemented before the crop is sown. Mid-season crop adaptation mechanisms expressed in response to stress, resource abundance, or other factors are potentially useful in regions where seasons can be quite variable. Phenotypic plasticity (herein termed as crop plasticity) refers to the ability of a genotype to adapt (e.g., express a specific trait) in response to the environment (Laitinen and Nikoloski, 2019). In sub-optimal or otherwise unpredictable growing conditions, crop plasticity mechanisms are being explored as an avenue to maintain yields (Nicotra et al., 2010).

Capitalizing on crop plasticity potential could improve the stability of production in regions with high climatic risk (Berzsenyi and Tokatlidis, 2012; Mylonas et al., 2020). The central US corn belt, where climate is relatively stable year to year, is an important hub of modern corn improvement. In this environment, breeders selected for those plasticity mechanisms conducive to high-yielding, intensively managed environments. Furthermore, as growers have intensified plant density and breeders have enhanced genetic tolerance to increased plant density over time, the expression of corn plasticity may have been constrained (Russell, 1991; Duvick et al., 2004; Assefa et al., 2018). When corn is sown at plant densities below those evaluated by breeding programs, alternative plasticity mechanisms, such as tillering, can be expressed (Lyon, 1905; Jenkins, 1941). Such plant densities (< 60000 plants ha^-1^) are commonly targeted by producers in restrictive environments like the Central High Plains of the US, the southwestern Pampas of Argentina, as well as portions of Africa and Australia. In these restrictive environments, multiple ears (prolificacy) or greater kernels per ear (commonly “flex”) are generally viewed by producers as desirable plasticity mechanisms when seasons are desirable, but only when expressed on the main shoot.

Tillers are secondary vegetative shoots common in Poacea species such as wheat (*Triticum aestivum* L.), rice (*Oryza sativa* L.), and grain sorghum (*Sorghum bicolor* L. Moench). However, tillers are less common in corn due to historic breeding selection (Major, 1977; Duvick et al., 2004). In spite of this, tiller expression potential has been conserved in modern corn germplasm (Moulia et al., 1999) and breeding program adoption of less restrictive plant densities re-introduces tillering as a plasticity mechanism (Tsaftaris et al., 2008). Tiller expression is highly dependent on genetics (Dungan et al., 1959; Tokatlidis et al., 2005; Hansey and de Leon, 2011), but also strongly influenced by environmental factors. Expressed corn tillers may remain vegetative, may abort, or may reach reproductive stages (Alofe and Schrader, 1975; Russelle et al., 1984) – developing into harvestable axillary ears or abnormal, mixed-sex apical inflorescences called “tassel ears” (Schaffner, 1930; Bonnett, 1948). Tiller development is ultimately a response to an abundance of resources, which may be triggered by nutrients, water, light, temperature, or factors resulting from a combination of these (e.g., plant density; Gardner, 1942; Downey, 1972; Stevenson and Goodman, 1972; Tetio-Kagho and Gardner, 1988). Although tiller development impacts are not well-documented in corn, yield of tillers has been proposed as respondent to factors such as plant density (light environment) and soil moisture (Thapa et al., 2018; Rotili et al., 2021; Veenstra et al., 2021).

While previous field studies have considered corn yield as a response to tiller presence (Sangoi et al., 2009; Frank et al., 2013; Veenstra et al., 2021; Massigoge et al., 2022), efforts to understand the mechanisms and flexibility of observed compensatory relationships are lacking, at least in the US. Considering trends in corn genetic selection and agronomic management in the US, plant density is a historic focal point (Duvick et al., 2004) with highly determinate, hierarchical yield components. Yield component plasticity (namely ears and kernels per area and individual kernel weight) in the idealized, single-stalked corn phenotype is marginal relative to the yield gain of additional plants per area (Fernández et al., 2022). For example, kernel number can be adjusted through early grain-filling stages but is limited by the success of a short pollination window (R1, silking per Ritchie et al., 1997) and the number of ears on the main stalk, which is determined in vegetative stages and typically singular (Bonnett, 1948; Andrade et al., 1999). Plastic phenotypes can reduce dependency on precise plant density (Tokatlidis and Koutroubas, 2004; Berzsenyi and Tokatlidis, 2012) – for instance, by producing more than one ear per plant (Prior and Russell, 1975; Thomison and Jordan, 1995). Tillers, a demonstrated source of plasticity, may facilitate an offset in development from the deterministic, single-stalked hierarchy. Functional trade-offs in resource allocation due to tiller expression are unknown. These relationships may improve or degrade yield stability.

Exploring the impact of tiller expression on yield component plasticity is a novel avenue to understand corn environmental adaptation potential. Although trade-offs in corn yield components are well-known (Slafer, 2003; Sadras and Slafer, 2012) and the concept of tiller-conferred plasticity has been established (Downey, 1972; Yamaguchi, 1974; Rotili et al., 2021; Rotili et al., 2022), field-based research solidifying the connection between the two is inadequate. Understanding the degree to which tillers impact reproductive plasticity may provide insight for reducing plant density dependence and shed new light on environmental adaptation strategies, particularly as climatic risk intensifies. A range in favorable to negligible yield responses to tiller expression were reported for the first two seasons of this project (Veenstra et al., 2021). Authors hypothesized that tiller expression improved plasticity of yield components, thereby reducing plant density-based yield dependency. Key points to explore in the dissection of observed yield responses included which yield components were most stabilized by tiller expression, if plasticity relationships were adjusted among yield components, and if yield component source (i.e., coming from tillers or main stalk) impacted yield stability and determination. Therefore, the aim of this investigation was to explore the consequences of tiller expression on corn yield component determination and plasticity in a range of environments from two plant fraction perspectives – i) main stalks only, considering potential functional trade-offs due to tiller expression; and ii) comprehensive (main stalk plus tiller contributions as an overall view of plasticity potential).

## 2 Materials and methods

### 2.1 Field experiments

**Figure 1:**
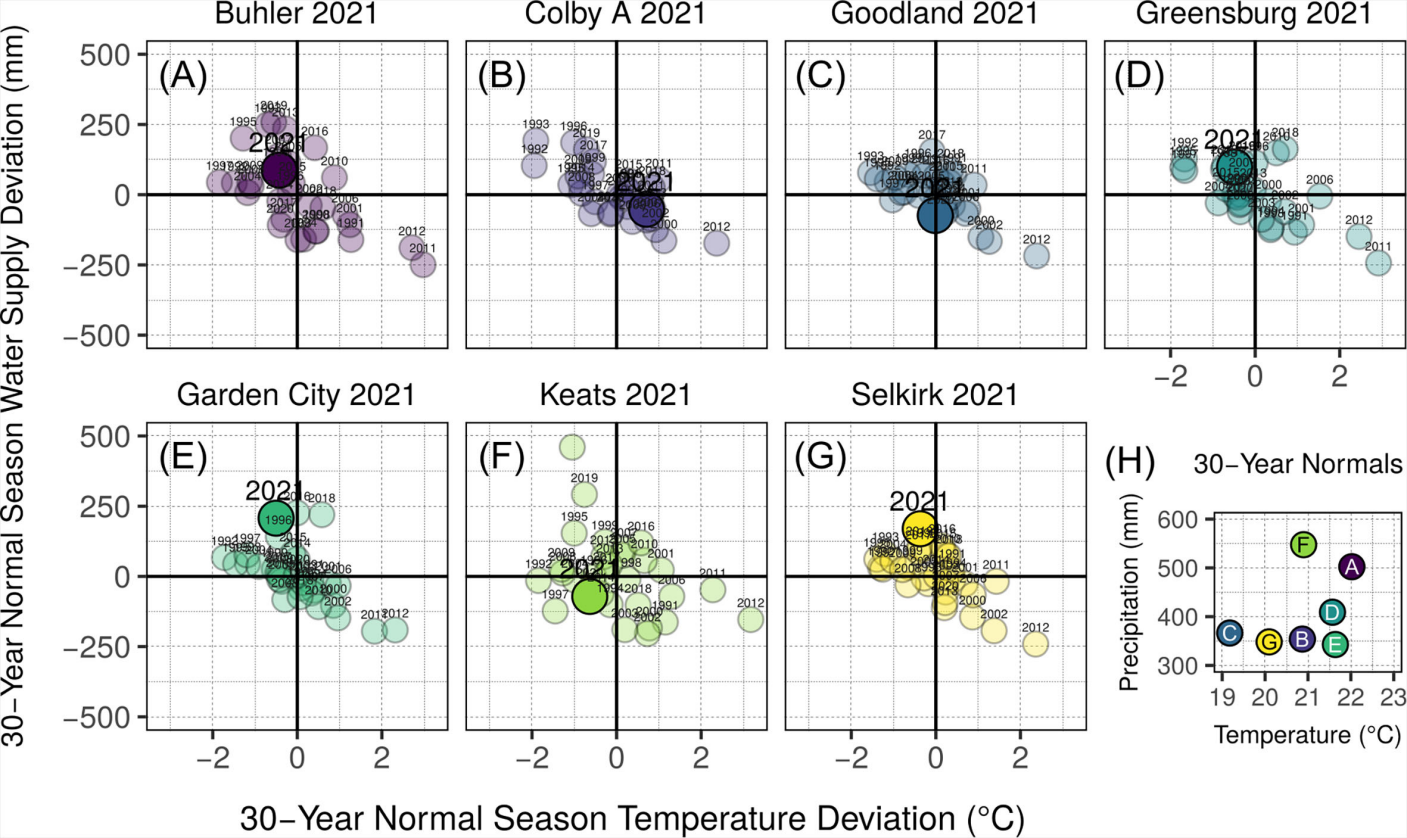
Environmental characterization of site-years added to those described previously in Veenstra et al., 2021. Annual season normal precipitation and temperature deviation for 1991-2020 are presented for each site-year **(A-G)**. Season normal precipitation and temperature characterization by site-year are shown in panel **(H)**, referring to the panel letter of described site-years. Bold vertical lines in panels **A—G** indicate normal average temperature for site-year season date ranges, while bold horizontal lines indicate normal precipitation accumulation for site-year season date ranges. Year of study for each site-year **(A-G)** is indicated with a large, opaque point and enlarged text, and considers both precipitation and irrigation in the water supply value (y-axis). All other years in panels **(A-G)** are shown with transparent points and smaller text, and water supply (y-axis) includes only precipitation. Base period for all climate normal calculations was 1991-2020.

Field trials were established at 9 sites across 3 years in Kansas, US, resulting in a final field database of 17 site-year combinations. In addition to the ten site-years (2019-2020 seasons) described in previous work (Veenstra et al., 2021), seven site-years were evaluated during the 2021 growing season. These 2021 site-year characterizations are provided in **Figure 1** and **Supplementary Table 1**. Sites ranged in seasonal normal precipitation from 330 to 550 mm and seasonal normal temperature from 19 to 22.5 °C (1991-2021 base period). Site coordinates ranged from 37.6 to 39.4 °N and 96.6 to 101.8 °W.

Of the full dataset, ten site-years were implemented using a replicated three-way factorial treatment structure in a randomized complete block design (RCBD) with a split-split-plot arrangement. Plant density was the whole plot factor, with three levels selected as representative of common producer practices in limited moisture environments across Kansas (25000, 42000, and 60000 plants ha^-1^; Roozeboom et al., 2007). Corn genotype (hybrid) was the sub-plot factor, with the two levels P0657AM and P0805AM (Corteva Agriscience, Johnston, IA, US), which were selected as suitable for the region of study and conducive to tiller. Both hybrids tillered at similar rates based on plant density, ranging from 0 (higher plant density) to 4.6 tillers plant^-1^ (lower plant density), with a mean of 0.8 tillers plant^-1^ for plots with tillers intact. Tiller presence was the sub-sub-plot factor, with the two levels intact or removed at development stage V10 (tenth leaf per Ritchie et al., 1997). Seven site-years were implemented with a similar RCBD design but missing either partial or total levels of the aforementioned treatment structure. Plots in all site-years were planted at least four rows wide at 0.76-m spacing, resulting in final minimum plot dimensions of 3 m by 5 m. Plant densities were seeded at double rates and thinned by hand prior to the V3 development stage to ensure accurate and even stands. Plant health was maintained as necessary with pesticides and crop nutritional needs were met with applied fertilizers.

Actual plant density, tiller density, and yield component data were collected at physiological maturity (development stage R6). Only the two central rows in each plot were included in data collection efforts. In addition, buffer zones were established on row ends to minimize edge effects. Tillers with at least one collared leaf were included in tiller density counts. Data rows were measured by carefully accounting for interplant spacing of the nearest buffer-appointed plant on row ends. Intact ears (machine-harvestable and providing > 100 collective kernels plot^-1^) were counted, picked, and shelled by hand at dry maturity (< 200 g kg^-1^ moisture). Data collected were summarized by plot but separated based on plant fraction – main stalk and tillers. Harvested areas across sites were similar in size and approximately 4 m by 1.5 m. Recorded yield components included ear number per area, kernel number per area, and weight per kernel. Kernel weights were measured with a representative sample of shelled grain for each plant fraction from each plot. Two sets of 100 kernels were counted and weighed, with values averaged, and final moisture content adjusted to a 155 g kg^-1^ basis. Kernels per area were calculated based on mean kernel weights and yield values. Plot averages for yield components were calculated as weighted means of main stalk and tiller data.

### 2.2 Calculations

Yield environment was previously linked to corn plasticity potential and tiller productivity (Rotili et al., 2021; Veenstra et al., 2021). Therefore, yield environment clusters were identified and characterized. Site-years were clustered by mean yield using the k-means algorithm. Based on the within-cluster sums of squares generated by the k-means algorithm, the ideal number of yield clusters was visually identified as three – low, moderate, and high. Soil texture and fertility were characterized via early season soil sampling at 15-cm and 60-cm depths. Plasticity was calculated with the methods used by Dingemanse et al. (2010) previously adapted for agronomic applications (Sadras and Rebetzke, 2013). According to this method, plasticity for each variable of interest (yield, for example) was calculated by dividing the variance of each hybrid in a given site-year by the variance of all observations in the study.

### 2.3 Statistical analysis

#### 2.3.1 Yield component response

All analyses were conducted using program R (R Core Team, 2022). Separate analyses were conducted for each yield component (ears per area, kernels per area, and kernel weight) considering i) main stalks only and ii) comprehensive plants. Initial treatment factor analyses were preformed first to discern if yield components responded to tiller presence. These initial analyses considered the ten site years with complete treatment structures. Ears per area, kernels per area, and kernel weight were each considered as a response to treatment factors plant density, genotype, and tiller presence for main stalks (ears and kernels harvested from main stalks only) and comprehensive plants (all ears and kernels harvested). Linear mixed effects models (**Supplementary Equation 1**) were fit to each yield component using the *lme4* package (Bates et al., 2015). All treatment factors and interactions were set as fixed effects. Random effects considered site-year, block, whole plot, and sub-plot. As only 10 of the 17 site years were implemented with a full split-split-plot structure and useful for initial analyses, study-wide yield environment cluster was not included in these models. The fitted models were subjected to a type III analysis of variance (ANOVA) for each treatment factor and resulting interactions with the *car* package (Fox and Weisberg, 2019).

Following analyses considered ears per area, kernels per area, and kernel weight as the response to observed plant density and observed tiller density for main stalks and comprehensive plants by yield environment, as all 17 site-years were included. Linear mixed effects models included fixed effects observed plant density, observed tiller density, yield environment cluster, and all two- and three-way interactions, in addition to random effects site-year, block, whole plot, and sub-plot (**Supplementary Equation 2**). The fitted models were subjected to a type III ANOVA for each factor and resulting interactions. Ears per area, kernels per area, and kernel weight predictions were generated using the significant fixed effect coefficient estimates from each of the fitted models. Predictive limits were identified based on observed ranges (20000 to 65000 plants ha^-1^ and 0 to 80000 tillers ha^-1^) and standardized across all environments. To maintain realistic perspective of tiller expression limits within each environment (i.e., not all environments produced similar tiller density trends), a third order polynomial regression was conducted with the 95^th^ percentile of tiller densities for each target plant density in each yield environment. This provided a plausible maximum observed tiller density for prediction interpretation purposes. Error was quantified with the root mean squared error (RMSE).

#### 2.3.2 Trait-yield plasticity relationships

To test correlation of yield plasticity with trait/yield component plasticity, simple linear models (*y* = *mx* + *b*) were fit using the *lm* function of the base *stats* package. Tiller number, ear number, and kernel number traits were evaluated by yield environment, as informed by prior analyses. Only plots without tiller disturbance were considered for this portion of the analyses. Appropriate models were selected separately for each yield environment with a slope parameter threshold of *p* ≤ 0.05.

#### 2.3.3 Yield response to ear type

To evaluate the relative importance of ear type (as a subset of yield component ear number) to maximizing yields, a linear mixed effects model was fit with grain yield (Mg ha^-1^) as the response variable. Fixed effects included observed main stalk primary ears ha^-1^, observed main stalk secondary ears ha^-1^, observed tiller axillary ears ha^-1^, and observed tiller apical ears (“tassel ears”) ha^-1^ by yield environment. Random effects considered site-year, block, whole plot, and sub-plot. The fitted model was subjected to a type II ANOVA for each ear type × environment combination. Error was quantified via the RMSE. Resulting yield predictions were generated using the significant fixed effect coefficient estimates. Predictive limits were identified based on observed ranges of ear types for each yield environment (primary, 16000 to 65000 ears ha^-1^; secondary, 0 to 43000 ears ha^-1^; tiller axillary, 0 to 43000 ears ha^-1^; and tiller apical, 0 to 31000 ears ha^-1^). The 95% confidence intervals were generated for each coefficient to check for similarities and overlaps.

## 3 Results

### 3.1 Yield environments

**Figure 2:**
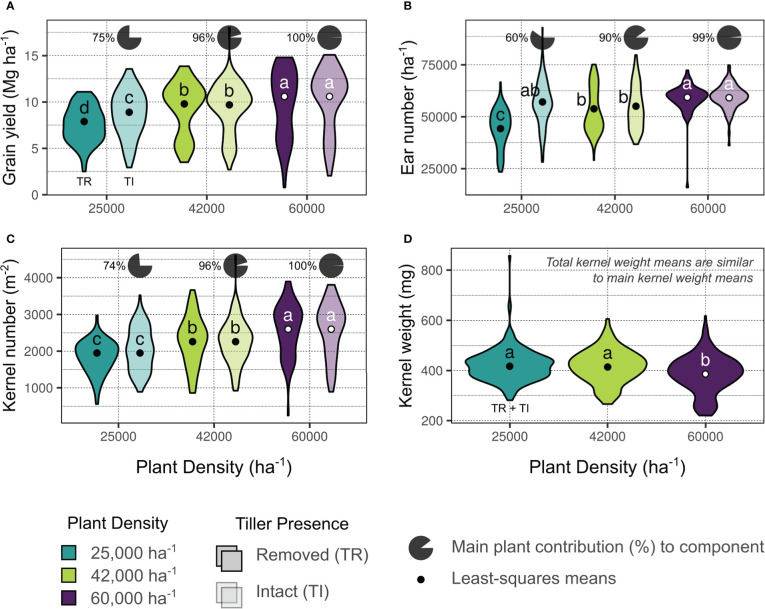
Summary of comprehensive yield **(A)**, and yield components (**(B)**, ear number; **(C)**, kernel number; **(D)**, kernel weight) based on treatment factors deemed significant by analysis of variance (**Supplementary Tables 2-4**). Colors indicate plant density (blue – 25000 plants ha^-1^, green – 42000 plants ha^-1^, purple – 60000 plants ha^-1^) and transparency indicates tiller presence (removed, TR – opaque; intact, TI – transparent). Data distribution is shown as a a violin plot and least-squares means from fitted models are indicated with points. Different letters indicate mean differences within each panel at the 0.05 probability level. Pie charts above TI plots indicate the percent contribution of main shoots to the comprehensive components (e.g., 75% of yield was produced by main shoots in TI plants at the 25000 plants ha^-1^ density, panel A).

The three yield environment clusters for all evaluated site-years were as follows: a) Lowest-yielding environments (LYEs) – Manhattan 2019, Colby B 2020, Colby A 2021 (mean 5.6 Mg ha^-1^); b) Moderate-yielding environments (MYEs) – Garden City 2019 and 2021, Buhler 2020 and 2021, Colby A 2020, Greensburg 2021 (mean 9.2 Mg ha^-1^); and c) Highest-yielding environments (HYEs) – Goodland 2019 through 2021, Garden City 2020, Greensburg 2020, Keats 2020 and 2021, Selkirk 2021 (mean 11.4 Mg ha^-1^). Grain yields across environments by treatment factors, with relative tiller contributions, are shown in **Figure 2A**. Tillers averaged 25% of total yield at 25000 plants ha^-1^.

### 3.2 Ears per area

The ANOVA results for models considering ears per area (ears ha^-1^) as a response are shown in **Figure 2B** and **Supplementary Table 2**. Both ears ha^-1^ responses (main stalks and comprehensive plants) were influenced by treatment factors plant density, tiller presence, and their interaction (all significant at *p* ≤ 0.001). Ears ha^-1^ ranged from 50703 to 59255 across target plant densities (25000 and 60000 plants ha^-1^, respectively), and increased 8% overall when tillers were present. The greatest ears ha^-1^ were observed in the 60000 plants ha^-1^ density with tillers removed (59348 ears ha^-1^), although this was not statistically different from the observed ears ha^-1^ in the 60000 or the 25000 plants ha^-1^ densities with tillers intact (59161 and 57114 ears ha^-1^, respectively). Tiller ears averaged 40% of total ears produced at 25000 plants ha^-1^ (**Figure 2B**).

**Figure 3:**
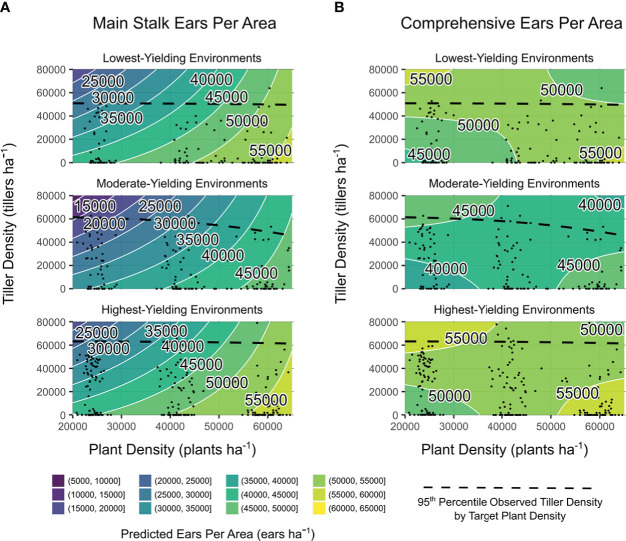
Main stalk ears per area **(A)** and comprehensive ears per area **(B)** predictions from models of observed plant density, tiller density, and yield environment as determined by analysis shown in **Supplementary Table 2**. Site-years are grouped by realized yield environment. Contours are shaded and labeled according to 5000 ears ha^−1^ density intervals. White lines indicate a change in ear density interval. Observed plant densities and tiller densities are indicated with black points. Black dashed lines are intended as an informal visual indicator of tiller expression potential for each yield environment. Extrapolations beyond black points and dashed black lines are shown for the purpose of comparing environments on the same density scales.

Additionally, both ears ha^-1^ responses were impacted by quantitative factors observed plant density and yield environment (*p* ≤ 0.001), observed tiller density (*p* ≤ 0.001, main; *p* ≤ 0.01, comprehensive), and the interaction between yield environment and observed plant density (*p* ≤ 0.01). Observation-based predictions for ears ha^-1^ are shown in **Figure 3**. Increased tiller densities reduced main stalk ears ha^-1^ in all yield environments, although less sharply at higher plant densities (**Figure 3A**). Plant density accounted for 50% of the predicted range in main stalk ears ha^-1^. Comprehensive ears ha^-1^ were more stable than main ears ha-1 regardless of tiller or plant densities (**Figure 3B**). Higher tiller densities reduced the plant density-based deficit in comprehensive ears ha^-1^. Greatest comprehensive ears ha^-1^ was predicted at both i) high observed plant densities with low observed tiller densities (all environments) and ii) low observed plant densities with high observed tiller densities (MYEs and HYEs).

### 3.3 Kernels per area

The ANOVA results for models considering kernels per area (kernels m^-2^) as a response are shown in **Figure 2C** and **Supplementary Table 3**. Main stalk kernels m^-2^ were influenced by treatment factors plant density and tiller presence (*p* ≤ 0.001), and their interaction (*p* ≤ 0.05). Comprehensive kernels m^-2^ were only impacted by plant density (p ≤ 0.001). Kernels m^-2^ ranged from 1950 (25000 plants ha^-1^) to 2599 (60000 plants ha^-1^). Tiller kernels averaged 25% of total kernels produced at 25000 plants ha^-1^ (**Figure 2C**).

**Figure 4:**
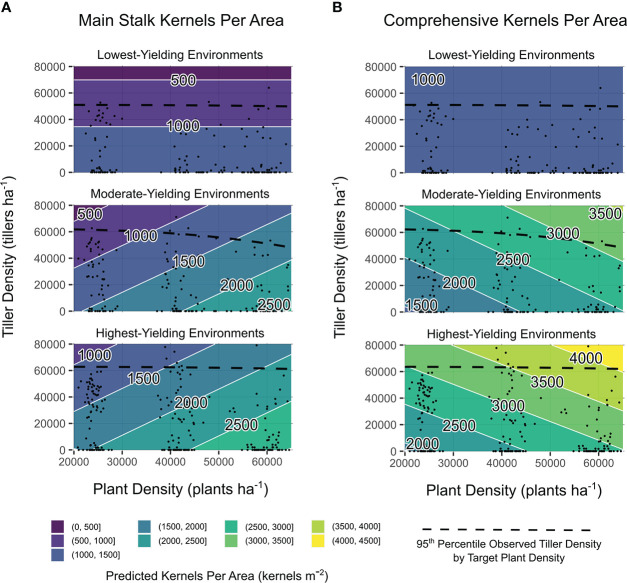
Main stalk kernels per area **(A)** and comprehensive kernels per area **(B)** predictions from models of observed plant density, tiller density, and yield environment as determined by analysis shown in **Supplementary Table 3**. Site-years are grouped by realized yield environment. Contours are shaded and labeled according to 500 kernels m^−2^ density intervals. White lines indicate a change in kernel density interval. Observed plant densities and tiller densities are indicated with black points. Black dashed lines are intended as an informal visual indicator of tiller expression potential for each yield environment. Extrapolations beyond black points and dashed black lines are shown for the purpose of comparing environments on the same density scales.

Main stalk kernels m^-2^ were influenced by quantitative variables tiller density, yield environment, and the interaction between yield environment and observed plant density (all significant at *p* ≤ 0.001). Comprehensive kernels m^-2^ were impacted by yield environment and the interaction between yield environment and observed plant density (*p* ≤ 0.001), in addition to the interaction between yield environment and observed tiller density (*p* ≤ 0.05). Considering observation-based predictions, increased tiller densities consistently reduced main stalk kernels m^-2^, regardless of plant density (**Figure 4A**). Plant density accounted for up to 75% of the range in predicted main stalk kernels m^-2^ when tillers were not present (**Figure 4A**). Comprehensive kernels m^-2^ were either not impacted by observed plant densities or tiller densities (LYEs) or independently influenced by both observed plant densities and tiller densities (MYEs and HYEs; **Figure 4B**). Greatest kernels per area were predicted at high observed plant densities with high observed tiller densities.

### 3.4 Kernel weights

The ANOVA results for models considering kernel weight (mg kernel^-1^) as a response are shown in **Figure 2D** and **Supplementary Table 4**. Main stalk kernel weight was influenced by treatment factors plant density (*p* ≤ 0.001), in addition to genotype and the interaction between plant density and tiller presence (*p* ≤ 0.05). Comprehensive kernel weights were impacted by treatment factors plant density (*p* ≤ 0.001) and genotype (*p* ≤ 0.05). Kernel weights ranged from 386 (60000 plants ha^-1^) to 417 (25000 plants ha^-1^) mg kernel^-1^ across plant densities. Genotypes differed in mean kernel weights by 10 mg kernel^-1^ (~2.4%; **Figure 2D**).

All kernel weight responses were influenced by quantitative factors observed plant density (*p* ≤ 0.05) and yield environment (*p* ≤ 0.001); predicted trends were similar between the two. Increased plant densities reduced both main stalk and comprehensive kernel weights in all environments, with a 25 to 50 mg kernel^-1^ discrepancy across observed plant densities. Trends were not impacted by tiller density and predictions are therefore not shown.

### 3.5 Trait-yield plasticity relationships

**Figure 5:**
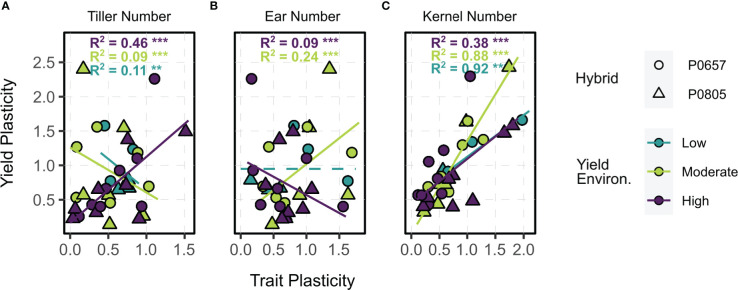
Relationships between trait plasticity (**A**, tiller number; **B**, ear number; **C**, kernel number) and yield plasticity (*y*) of tillered phenotypes. Points are colored by yield environment (blue – low, green – moderate, purple – high); and shaped by hybrid (circle – P0657AM, triangle – P0805AM). Fitted lines and model metrics, when applicable, are colored by yield environment. Dashed lines indicate intercept-only models when other candidates were not significant. Significance symbols are the following: ** *p* ≤ 0.01, *** *p* ≤ 0.001.

Tillered phenotype trait plasticity correlations with yield plasticity varied by yield environment (**Figure 5**). Tiller number plasticity (i.e., the situational nature of tiller expression in a given environment) reduced yield plasticity in LYEs and MYEs, ultimately acting to stabilize yields (**Figure 5A**). Greater plasticity of tiller number was associated with greater plasticity of yield in HYEs, however. Ear number plasticity reduced yield plasticity in HYEs, increased yield plasticity in MYEs, and had no impact on yield plasticity in LYEs (**Figure 5B**). Kernel number plasticity exhibited the strongest relationship to yield plasticity across environments, with greater plasticity of kernel number increasing yield plasticity (**Figure 5C**). That is, stable kernel numbers were the yield component most correlated with stable yield values in a given environment.

### 3.6 Ear type relationship to attainable yields

The ANOVA results for yield response to varying ear sources by yield environment are presented in **Supplementary Table 5**. The only ear source not significantly contributing (*p* > 0.05) to yield determination was tiller apical ears. This coefficient estimate was therefore not included in subsequent predictions.

**Figure 6:**
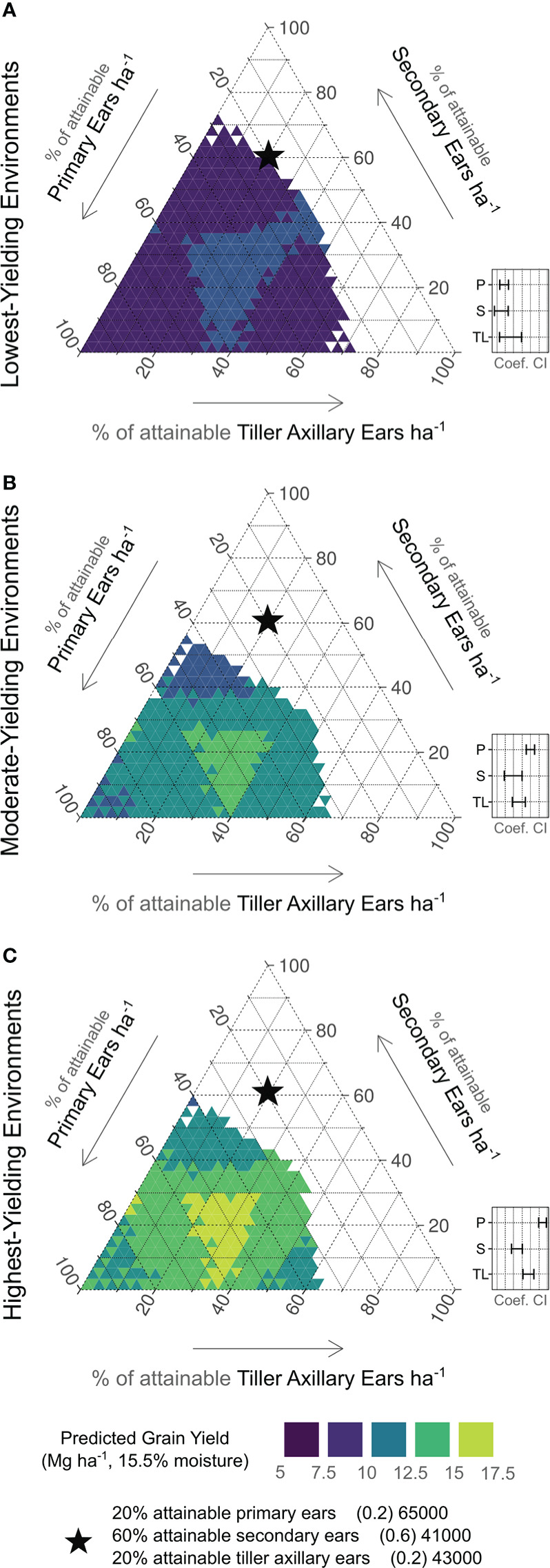
Ear type plasticity relationship to predicted yields by yield environment (**A**, low; **B**, moderate; **C**, high). Right axes indicate % of attainable primary ears observed (65000 ears ha^-1^). Left axes indicate % of attainable secondary ears observed (41000 ear ha^-1^). Bottom axes indicate % of attainable tiller axillary ears observed (43000 ears ha^-1^). Contour shades indicate predicted yield level (purple < 7.5 Mg ha^-1^; lime > 15 Mg ha^-1^). Black star is shown for reference on each plot, indicating 20%, 60%, and 20% of attainable primary ears, secondary ears, and tiller axillary ears, respectively. Insets show relative 95% confidence intervals for each coefficient estimate (P, primary ears; S, secondary ears; TL, tiller axillary ears).

Yield predictions based on various combinations of ear types by yield environment are shown in **Figure 4**. In these ternary plots, each axis depicts the % of attainable ears ha^-1^. The 95% confidence intervals for coefficient estimates are presented as insets. In LYEs (**Figure 6A**), predicted yields were greatest with 17 to 67% of attainable primary ears (11050 to 43550 ears ha^-1^), 0 to 50% of attainable secondary ears (0 to 20500 ears ha^-1^), and 0 to 50% of attainable tiller axillary ears (0 to 21500 ears ha^-1^). Confidence intervals overlapped for all ear types in LYEs, indicating one ear type was not more effective in producing yields than others.

In MYEs (**Figure 6B**), predicted yields were greatest with 37 to 77% of attainable primary ears (24050 to 50050 ears ha^-1^), 0 to 30% of attainable secondary ears (0 to 12300 ears ha^-1^), and 0 to 40% of attainable tiller axillary ears (0 to 17200 ears ha^-1^). The lowest predicted yields in MYEs were most associated with greater than 40% of attainable secondary ears. Confidence intervals indicated that primary ears exceeded other ear types in producing yield, but secondary and tiller axillary ears remained similar to each other.

In HYEs (**Figure 6C**), predicted yields were greatest with 37 to 77% of attainable primary ears (24050 to 50050 ears ha^-1^), 0 to 30% of attainable secondary ears (0 to 12300 ears ha^-1^), and 0 to 40% of attainable tiller axillary ears (0 to 17200 ears ha^-1^). The lowest predicted yields in HYEs were most associated with > 40% of attainable secondary ears, > 50% of attainable tiller axillary ears, and > 80% of attainable primary ears ha^-1^. Considering 95% confidence intervals, a more distinct hierarchy was evident compared to other environments (primary ears > tiller axillary ears > secondary ears) in yield formation.

## 4 Discussion

This study advances corn plasticity discussions by considering the unexplored extent of tiller compensatory relationships across contrasting environments and management practices (particularly plant density). Authors present data on yield component determination in tillered corn phenotypes from both main stalk and comprehensive plant perspectives in field-scale trials, thereby filling a deficit in available literature. Findings from this study apply to a considerable range of environment × management conditions, as the dataset included 17 unique site-years covering typical plant density ranges in environments similar to the semi-arid US High Plains. These 17 site-years, including the 10 site-years previously analyzed for simple yield relationships (without considering yield components) in (Veenstra et al., 2021), represent a significant, detailed corn tiller expression field study database. The tillering element of corn physiology is actively being studied at a global scale, with authors utilizing both simulation and in-field approaches to understand key mechanisms and utility (Rotili, Sadras, et al., 2021; Veenstra et al., 2021). In agreement with published literature, evaluated field trials demonstrate that tiller expression facilitates crop plasticity in response to resource availability with favorable genetics (Jenkins, 1941). Tiller appearance and development mechanisms were not explored in the current study, which limits discussion scope to reproductive outcomes (evaluated yield components). A key caveat of this study is the fact that producers will not intentionally manage corn fields to promote tillering. The results shown here consider a case in which the optimum plant density is unknown or not achieved – either due to stress, damage, or a more resourceful season than anticipated at planting (i.e., too conservative plant density selected). Tillering may have some benefits in such a scenario. This exploration of plasticity mechanisms is critical to understanding how crops (even relatively determinate ones, like corn) can cope with shifts in environment.

Flexible tiller densities were associated with more stable yield component predictions across all environments. This physiological response is of particular interest when seasonal resources are more abundant early in the growing season (Veenstra et al., 2021) to reduce dependence on plant density (Berzsenyi and Tokatlidis, 2012). Considering the density-dependent nature of yield progress in breeding and management of modern corn hybrids, this result is not surprising in tillered phenotypes (Duvick et al., 2004). An optimized plant density remained critical to maximize ear number, which supports the yield observations in previous tiller response work (Veenstra et al., 2021). However, kernel number was maximized with greater tiller development across plant densities in the present study. The modeled corn tiller expression scenarios of Rotili et al. (2021) indicated changes in kernels per area due to tillering were determined by yield environment, with marginal environments experiencing reductions in kernel number. In the present study, however, tiller density was only neutral or additive to total kernels per area. This difference is perhaps tied to the more restrictive environments evaluated by Rotili, Abeledo, et al., but it should be noted that both studies predicted/observed similar ranges of kernel set (1000 to 3000 kernels m^-2^).

Although main stalk ears and subsequent kernels per area were reduced in lower plant densities with tiller expression, kernel weights remained relatively stable regardless of tiller expression. While these results may suggest main stalk yield reductions, work by Veenstra and Ciampitti (2021) indicated that tiller presence did not significantly reduce main stalk grain yields in the same environments considered in the present study. The lack of tiller expression impact on main stalk kernel weights also supports the hypothesis of an independent (i.e., grain-bearing tillers in lower plant densities) or nourishing (i.e., non-reproductive tillers in higher plant densities) energy and nutrient remobilization relationship for tillers and main stalk during late-season yield determination (Alofe and Schrader, 1975; Russelle et al., 1984). A key function of tillers is increasing leaf area and aboveground biomass, thus enhancing the source of energy through photosynthates and grain set potential (Lafarge and Hammer, 2002). Essentially, tillers may 1) contribute to yield directly, 2) increase the rate of light interception and growth, and/or 3) store remobilizable carbon and nitrogen. This point of source-sink relationships in tillered phenotypes requires further investigation.

Kernel number was the most significant component related to yield plasticity across all environments. This result is not surprising, as kernel number is known to be key to corn yield determination (Andrade et al., 1999). In general, situational tiller expression could be associated with non-uniform field features, which is a yield-negating factor for intensively managed corn (Hörbe et al., 2016). Tillering increased corn kernel numbers for shoots with high growth rates in field studies conducted by Rotili et al. (2022). Corn growth rates required to set kernels on primary ears appear to be lower than for tillers, and low growth rates are associated with stressed conditions (Andrade et al., 1999). In this regard, authors note that evaluated conditions in the present study may not have been harsh enough to observe such a response.

Ear number was less significantly related to yield stability than kernel number and varied by environment, which may be due to the potentially abnormal nature of tiller reproductive development (Ortez et al., 2022). A key determinant of tiller contributions to kernel number is successful reproductive development of tillers (i.e., pollination and grain fill of axillary ears). Although tiller apical ears were not found to be significant to corn yields in this study, tiller axillary ears were quite relevant, even when secondary ears were present on the main stalk. While main stalk prolificacy is commonly presented as a source of corn plasticity in environments where low plant densities are employed, secondary ears were found to be a slightly weaker source of yield than tiller axillary ears in the best-yielding environments. Similar relationships between tiller axillary and secondary ears kernel number were observed in some cases by Rotili et al. (2022). Such findings indicate value in diversifying yield determination hierarchy with tiller ears in some cases. Additionally, main stalk prolificacy has obvious limits (Tokatlidis et al., 2005; Mylonas et al., 2020), and the presented results identify tiller utility when these limits are realized. Key to note, however, are the low predicted yields when too many tiller axillary ears were present, reaffirming the importance of optimized plant densities in HYEs (Veenstra et al., 2021). Previous studies have suggested that tillering reduces yield efficiency (Kapanigowda et al., 2010; Thapa et al., 2018), but this blanket hypothesis was recently rejected (Rotili et al., 2022). Additional exploration of tiller reproductive development (i.e., vegetative, axillary ear, or apical ear) and potential impacts on efficiency metrics is needed.

While continued study is necessary, corn tillers may provide breeders and growers with plasticity trait options to achieve desirable plant density independence in certain environments (Mylonas et al., 2020). By offering additional crop reproductive plasticity when plant-available resources surpass thresholds of selected plant densities, tillers can mitigate management deficits which cannot be remediated mid-season (Rotili et al., 2021; Veenstra et al., 2021; Massigoge et al., 2022). Future work should evaluate tiller development prediction, specifically driving factors of contrasting levels of expression plasticity, in addition to parameters influencing tiller ear development and resulting reproductive efficiency.

## Data availability statement

The raw data supporting the conclusions of this article will be made available by the authors, without undue reservation.

## Author contributions

RV: Conceptualization, Investigation, Project Administration, Methodology, Formal Analysis, Visualization, Writing – original draft, Writing – review and editing. CM: Conceptualization, Methodology, Writing – review and editing. DB: Methodology, Writing – review and editing. LH: Resources, Investigation. PC: Conceptualization, Methodology, Writing – review and editing. TH: Writing – review and editing. PP: Writing – review and editing. IC: Conceptualization, Methodology, Supervision, Project Administration, Funding Acquisition, Writing – original draft, Writing – review and editing. All authors contributed to the article and approved the submitted version.
