# Supplementary material for: Corn yield components can be stabilized via tillering in sub-optimal plant densities
Source: Front Plant Sci. 2023 Jan 6;13:1047268. doi: 10.3389/fpls.2022.1047268 (PMC9853411; doi:10.3389/fpls.2022.1047268)
Supplement: Supplementary file 1 [file Presentation_1.pdf]

frontiers

Supplementary Material

Supplementary Table 1. Site-year field experiment coordinates, sow date, tenth-leaf (V10) date, treatment structure (D, Density; G, Genotype; P, Tiller Presence), irrigation, previous crop, and soil characterization [pH, organic matter (OM – loss on ignition), nitrate concentration (NO_3-N), ammonium concentration (NH_4-N), phosphorus (P – Mehlich), cation exchange capacity (CEC), and soil texture]. Only the 7 additional site-years to the 10 already described in Veenstra et al. (2021) are shown here.

Site-Year	Latitude	Longitude	Sow Date	V10 Date	Treatment Structure	Irrigation	Previous Crop	pH	OM	NO3-N	NH4-N	P	CEC	Soil Texture
	(°N)	(°W)						($H_2$0)	% (LOI)	(mg kg^{-1})	(mg kg^{-1})	Mehlich (mg kg^{-1})	(meq 100g^{-1})	
Keats 2021	39.23	96.72	Apr-30	Jun-22	D x G x P	None	Maize	6.6	6.2	23.3	12.7	106.4	25.6	Silt Loam
Buhler 2021	38.14	97.73	May-04	Jun-25	D x G	Subsurface limited	Maize	6.3	2.6	11.7	7.8	13.3	22.3	Silt Loam
Greensburg 2021	37.58	99.37	May-07	Jun-25	D x G	Subsurface limited	Maize	5.6	2.3	33.4	7.4	68.8	20.0	Loam
Selkirk 2021	38.70	101.54	May-06	Jun-30	D x G	Subsurface limited	Field Bean	7.9	2.7	14.0	5.8	90.9	23.2	Loam
Garden City 2021	37.83	100.86	May-13	Jun-28	D x G x P	Subsurface limited	Maize	5.5	1.6	14.2	5.2	52.1	9.7	Sandy Loam
Goodland 2021	39.25	101.78	May-05	Jun-30	D x G x P	Subsurface limited	Soybean	6.5	2.9	36.9	11.1	65.4	23.2	Loam
Colby A 2021	39.39	101.06	Jun-04	Jul-15	D x G x P	None	Wheat	7.1	2.9	23.8	7.1	93.0	22.2	Clay Loam

Supplementary Table 2. ANOVA results for ear density given treatment factors target plant density (D), genotype (G), tiller presence (P), and interactions; and observed variables main plant density (M), observed tiller density (T), yield environment (E), and interactions. Tested source of variation (Source), degrees of freedom (df), degrees of freedom of residuals (Residual df), F value, and the associated *p* value significance are presented. All sources with *p* values ≤ 0.05 are shown in boldface font. Coefficient of determination (R^2) and root mean square error (RMSE) values for model fit are presented below each section.

Response Variable	Source	df	Residual df	F value	*p* value
Main stalk ears ha^{-1} (treatment factors, 10 site-years)	**Plant Density (D)**	**3**	**49.59**	**263.29**	***
	Genotype (G)	1	264.00	0.02	ns
	**Tiller Presence (P)**	**1**	**198.00**	**55.68**	***
	D × G	2	264.00	0.92	ns
	**D × P**	**2**	**198.00**	**16.22**	***
	G × P	1	198.00	0.12	ns
	D × G × P	2	198.00	0.26	ns
	Marginal R^2 = 0.23, Conditional R^2 = 0.55, RMSE = 5360 ears ha^{-1}				
Comprehensive ears ha^{-1} (treatment factors, 10 site-years)	**Plant Density (D)**	**3**	**47.38**	**321.70**	***
	Genotype (G)	1	251.34	0.00	ns
	**Tiller Presence (P)**	**1**	**198.00**	**39.63**	***
	D × G	2	251.34	1.64	ns
	**D × P**	**2**	**198.00**	**10.83**	***
	G × P	1	198.00	0.96	ns
	D × G × P	2	198.00	0.32	ns
	Marginal R^2 = 0.52, Conditional R^2 = 0.77, RMSE = 6933 ears ha^{-1}				
Main stalk ears ha^{-1} (observed variables, 17 site-years)	**Observed Plant Density (M)**	**1**	**183.32**	**35.11**	***
	**Observed Tiller Density (T)**	**1**	**307.84**	**28.51**	***
	**Yield Environment (E)**	**3**	**82.72**	**146.17**	***
	**M × T**	**1**	**334.69**	**9.94**	**
	M × E	2	195.43	2.58	ns
	T × E	2	341.09	0.09	ns
	M × T × E	2	375.77	0.53	ns
	Marginal R^2 = 0.62, Conditional R^2 = 0.79, RMSE = 4892 ears ha^{-1}				
Comprehensive ears ha^{-1} (observed variables, 17 site-years)	**Observed Plant Density (M)**	**1**	**186.46**	**25.42**	***
	**Observed Tiller Density (T)**	**1**	**326.98**	**10.24**	**
	**Yield Environment (E)**	**3**	**92.78**	**127.19**	***
	**M × T**	**1**	**360.31**	**9.42**	**
	M × E	2	200.27	2.18	ns
	T × E	2	354.39	1.21	ns
	M × T × E	2	390.76	0.27	ns
	Marginal R^2 = 0.32, Conditional R^2 = 0.55, RMSE = 6386 ears ha^{-1}				

*** significant at $p \leq 0.001$, ** significant at $p \leq 0.01$, ns not significant

Supplementary Table 3. Analysis of variance results for kernel density given treatment factors target plant density (D), genotype (G), tiller presence (P), and interactions; and observed variables main plant density (M), observed tiller density (T), yield environment (E), and interactions. Tested source of variation (Source), degrees of freedom (df), degrees of freedom of residuals (Residual df), F value, and the associated *p* value significance are presented. All sources with *p* values ≤ 0.05 are shown in boldface font. Coefficient of determination (R^2) and root mean square error (RMSE) values for model fit are presented below each section.

Response Variable	Source	df	Residual df	F value	*p* value
Main stalk kernels m^{-2} (treatment factors, 10 site-years)	**Plant Density (D)**	**3**	**40.78**	**97.78**	***
	Genotype (G)	1	227.15	2.91	ns
	**Tiller Presence (P)**	**1**	**198.00**	**24.43**	***
	D × G	2	227.15	1.21	ns
	**D × P**	**2**	**198.00**	**4.60**	*
	G × P	1	198.00	0.08	ns
	D × G × P	2	198.00	0.03	ns
	Marginal R^2 = 0.31, Conditional R^2 = 0.84, RMSE = 243 kernels m^{-2}				
Comprehensive kernels m^{-2} (treatment factors, 10 site-years)	**Plant Density (D)**	**3**	**42.25**	**79.28**	***
	Genotype (G)	1	253.36	2.51	ns
	Tiller Presence (P)	1	198.00	2.75	ns
	D × G	2	253.36	1.08	ns
	D × P	2	198.00	1.85	ns
	G × P	1	198.00	1.70	ns
	D × G × P	2	198.00	0.64	ns
	Marginal R^2 = 0.15, Conditional R^2 = 0.77, RMSE = 307 kernels m^{-2}				
Main stalk kernels m^{-2} (observed variables, 17 site-years)	Observed Plant Density (M)	1	186.50	1.75	ns
	**Observed Tiller Density (T)**	**1**	**287.00**	**11.17**	***
	**Yield Environment (E)**	**3**	**108.57**	**74.14**	***
	M × T	1	306.28	3.77	ns
	**M × E**	**2**	**191.68**	**20.68**	***
	T × E	2	323.51	0.00	ns
	M × T × E	2	354.50	0.01	ns
	Marginal R^2 = 0.66, Conditional R^2 = 0.84, RMSE = 235 kernels m^{-2}				
Comprehensive kernels m^{-2} (observed variables, 17 site-years)	Observed Plant Density (M)	1	183.72	2.95	ns
	Observed Tiller Density (T)	1	315.22	0.09	ns
	**Yield Environment (E)**	**3**	**70.96**	**61.60**	***
	M × T	1	343.49	0.13	ns
	**M × E**	**2**	**196.35**	**20.57**	***
	**T × E**	**2**	**345.99**	**4.33**	*
	M × T × E	2	380.61	2.53	ns
	Marginal R^2 = 0.57, Conditional R^2 = 0.76, RMSE = 293 kernels m^{-2}				

*** significant at p ≤ 0.001, * significant at p ≤ 0.05, ns not significant

Supplementary Table 4. Analysis of variance results for kernel weight given treatment factors target plant density (D), genotype (G), tiller presence (P), and interactions; and observed variables main plant density (M), observed tiller density (T), yield environment (E), and interactions. Tested source of variation (Source), degrees of freedom (df), degrees of freedom of residuals (Residual df), F value, and the associated *p* value significance are presented. All sources with *p* values ≤ 0.05 are shown in boldface font. Coefficient of determination (R^2) and root mean square error (RMSE) values for model fit are presented below each section.

Response Variable	Source	df	Residual df	F value	*p* value
Main Stalk Kernel Weight (treatment factors, 10 site-years)	**Plant Density (D)**	**3**	**45.50**	**189.75**	*******
	**Genotype (G)**	**1**	**249.84**	**5.06**	*****
	Tiller Presence (P)	1	198.00	3.36	ns
	D × G	2	249.84	1.42	ns
	**D × P**	**2**	**198.00**	**3.16**	*****
	G × P	1	198.00	0.01	ns
	D × G × P	2	198.00	0.06	ns
	Marginal R^2 = 0.08, Conditional R^2 = 0.60, RMSE = 42.5 mg $kernel^{-1}$				
Comprehensive Kernel Weight (treatment factors, 10 site-years)	**Plant Density (D)**	**3**	**45.31**	**187.62**	*******
	**Genotype (G)**	**1**	**248.72**	**5.34**	*****
	Tiller Presence (P)	1	198.00	2.05	ns
	D × G	2	248.72	1.50	ns
	D × P	2	198.00	2.89	ns
	G × P	1	198.00	0.66	ns
	D × G × P	2	198.00	0.36	ns
	Marginal R^2 = 0.15, Conditional R^2 = 0.77, RMSE = 41.9 mg $kernel^{-1}$				
Main Stalk Kernel Weight (observed variables, 17 site-years)	**Observed Plant Density (M)**	**1**	**187.83**	**6.05**	*****
	Observed Tiller Density (T)	1	332.13	1.49	ns
	**Yield Environment (E)**	**3**	**89.95**	**388.45**	*******
	M × T	1	367.25	2.87	ns
	M × E	2	201.68	0.81	ns
	T × E	2	357.11	0.24	ns
	M × T × E	2	393.53	0.72	ns
	Marginal R^2 = 0.32, Conditional R^2 = 0.54, RMSE = 46.1 mg $kernel^{-1}$				
Comprehensive Kernel Weight (observed variables, 17 site-years)	**Observed Plant Density (M)**	**1**	**188.11**	**6.35**	*****
	Observed Tiller Density (T)	1	332.89	0.42	ns
	**Yield Environment (E)**	**3**	**88.59**	**420.86**	*******
	M × T	1	368.32	1.69	ns
	M × E	2	201.95	0.91	ns
	T × E	2	357.34	0.09	ns
	M × T × E	2	393.71	0.91	ns
	Marginal R^2 = 0.32, Conditional R^2 = 0.54, RMSE = 44.6 mg $kernel^{-1}$				

*** significant at p ≤ 0.001, * significant at p ≤ 0.05, ns not significant

Supplementary Table 5. Analysis of variance results for yield response given observed variables primary ears ha^{-1}, secondary ears ha^{-1}, tiller axillary ears ha^{-1}, and tiller apical ears ha^{-1} by yield environment (E). Tested source of variation (Source), degrees of freedom (df), degrees of freedom of residuals (Residual df), F value, and the associated *p* value significance are presented. All sources with *p* values ≤ 0.05 are shown in boldface font. Coefficient of determination (R^2) and root mean square error (RMSE) values for model fit are presented below each section.

Source	df	Residual df	F value	*p* value
**Primary Ears × Yield Environment (E)**	**3**	**119.67**	**568.12**	***
**Secondary Ears × E**	**3**	**527.00**	**38.69**	***
**Tiller Axillary Ears × E**	**3**	**524.34**	**81.03**	***
Tiller Apical Ears × E	3	522.37	2.30	ns
Marginal R^2 = 0.84, Conditional R^2 = 0.84, RMSE = 1.16 Mg ha^{-1}				

Supplementary Equation 1.

$$y_{ijklmnq} = \mu + \alpha_i + \beta_j + \theta_k + \delta_l + \alpha_i\beta_j + \alpha_i\theta_k + \alpha_i\delta_l + \beta_j\theta_k + \beta_j\delta_l + \theta_k\delta_l + \alpha_i\beta_j\theta_k + \alpha_i\beta_j\delta_k + \beta_j\theta_k\delta_l + \alpha_i\beta_j\theta_k\delta_l + b_m + d_{n(m)} + h_{q(n(m))} + \varepsilon_{ijklmnq},$$

$$b_m \sim \mathrm{N}(0, \sigma^2_b),$$

$$d_{n(m)} \sim \mathrm{N}(0, \sigma^2_d),$$

$$h_{q(n(m))} \sim \mathrm{N}(0, \sigma^2_h), \text{ and}$$

$$\varepsilon_{ijklmnq} \sim \mathrm{N}(0, \sigma^2_\varepsilon).$$

In this case, $y_{ijklmnp}$ is one of the observed yield component responses for site-years with a complete three-way factorial treatment structure, namely

- main stalk ear density (ears ha^{-1}),
- total ear density (ears ha^{-1}),
- main stalk kernel density (kern m^{-2}),
- total kernel density (kern m^{-2}),
- main stalk kernel weight (mg kern^{-1}), or
- total kernel weight (mg kern^{-1}),

of a plot with tiller presence level *k* of genotype *j* of target plant density *i* of site-year *l*, observed in sub-plot *q* of whole plot *n* of block *m*; μ is the overall mean (intercept); α_i is the fixed effect of the ith level of target density; β_j is the fixed effect of the jth level of genotype; θ_k is the fixed effect of the kth level of tiller presence; δ_l is the fixed effect of the lth site-year; all combinations of the factors α_i, β_j, θ_k, and δ_l indicate double, triple, and quadruple interactions between them at double, triple, and quadruple levels; b_m is the random intercept effect of block *m*; $d_{n(m)}$ is the random intercept effect of the whole plot within block *m*; $h_{p(n(m))}$ is the random intercept effect of the sub-plot within whole plot *n* within block *m*; and $\varepsilon_{ijklmnp}$ is the residual term.

Supplementary Equation 2.

$$y_{ijklmn} = \mu + \alpha_j + \beta m_i + \gamma t_i + \theta m_i t_i + \delta_j m_i + \tau_j t_i + \zeta_j m_i t_i + s_k + b_{l(k)} + d_{m(l(k))} + h_{n(m(l(k)))} + \varepsilon_{ijklmn},$$
$$s_k \sim N(0, \sigma^2_s),$$
$$b_{l(k)} \sim \mathrm{N}(0, \sigma^2_b),$$
$$d_{m(l(k))} \sim \mathrm{N}(0, \sigma^2_d),$$
$$h_{n(m(l(k)))} \sim \mathrm{N}(0, \sigma^2_h), \text{ and}$$
$$\varepsilon_{ijklmn} \sim \mathrm{N}(0, \sigma^2_\varepsilon).$$

In this case, y_{ijklmn} is one of the observed yield component responses, namely

- main stalk ear density (ears ha^{-1}),
- total ear density (ears ha^{-1}),
- main stalk kernel density (kern m^{-2}),
- total kernel density (kern m^{-2}),
- main stalk kernel weight (mg $kern^{-1}$), or
- total kernel weight (mg $kern^{-1}$),

of the ith plot, found in sub-plot n of whole plot m of block l in site-year k of environmental cluster j; m_i is the observed main plant density of plot i; t_i is the observed tiller density of plot i; μ is the overall mean (intercept); α_j is the fixed effect of the jth level of environmental cluster; β is the fixed effect associated with observed main plant density; γ is the fixed effect associated with observed tiller density; θ is the fixed effect associated with the interaction of observed main plant density and observed tiller density; δ_j is the fixed effect associated with observed main plant density in environment cluster j; τ_j is the fixed effect associated with observed tiller density in environment cluster j; ζ_j is the fixed effect associated with interaction of observed main plant density and observed tiller density within environment cluster j; s_k is the random intercept effect of site-year k; $b_{l(k)}$ is the random intercept effect of block l within site-year k; $d_{m(l(k))}$ is the random intercept effect of the whole plot within block l within site-year k; $h_{n(m(l(k)))}$ is the random intercept effect of the sub-plot within whole plot m within block l within site-year k; and ε_{ijklmn} is the residual term.

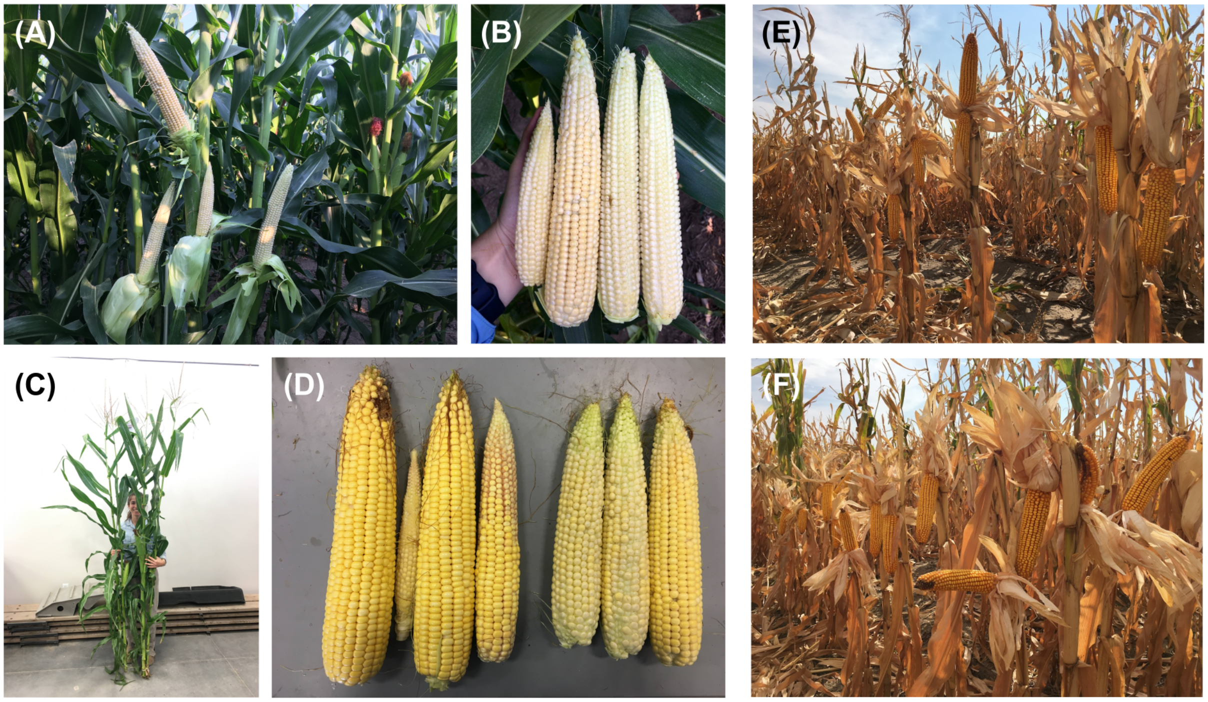

Supplementary Figure 1. Images of corn tillering experiment conducted in 2019-2021 seasons across Kansas, United States. Panel A – husked ears on a single plant with tillers intact in 42000 plants ha^{-1} density plot in Goodland, Kansas. Panel B – ears taken from plant shown in panel A (main shoot, left two ears; tillers, right two ears). Panel C – two tillered plants (one in each of person's arms) chosen as a representative sample of the 25000 plants ha^{-1} plots with tillers intact in Garden City, Kansas. Tillers are as tall or taller than the main shoot, which is on the left side of each plant. Panel D – ears taken from plant shown in panel C (main shoot, left four ears; tillers, right three ears). Panel E – central row of 25000 plants ha^{-1} plot with tillers removed (row in background removed same day for data collection purposes). Panel F – central row of 25000 plants ha^{-1} plot with tillers intact (row in background removed same day for data collection purposes).
